# Longitudinal Testing of Olfactory and Gustatory Function in Patients with Multiple Sclerosis

**DOI:** 10.1371/journal.pone.0170492

**Published:** 2017-01-20

**Authors:** Florian Cornelius Uecker, Heidi Olze, Hagen Kunte, Christian Gerz, Önder Göktas, Lutz Harms, Felix Alexander Schmidt

**Affiliations:** 1 Department of Otorhinolaryngology, Charité –Universitätsmedizin Berlin, Berlin, Germany; 2 Clinical and Experimental Multiple Sclerosis Research Center, Department of Neurology, Charité –Universitätsmedizin Berlin, Berlin, Germany; 3 MSB - Medical School Berlin, Berlin, Germany; Instituto Cajal-CSIC, SPAIN

## Abstract

**Background:**

The aim of the study was to investigate changes of the olfactory and gustatory capacity in patients with multiple sclerosis (MS).

**Methodology:**

20 MS patients were tested longitudinally for 3 years after initial testing. The Threshold Discrimination Identification test (TDI) was used for subjective olfactometry. Objective olfactometry was performed by registering olfactory evoked potentials (OEP) by EEG. The Taste Strip Test (TST) was used for gustatory testing.

**Results:**

45% of the patients showed olfactory dysfunction in the follow-up TDI test and 50% showed delayed OEP´s. 20% of the patients showed gustatory dysfunction on follow-up visit. The patients showed mild disease activity with 0,3 ± 0,5 relapses over the testing period and no significant change of their olfactory and gustatory capacity. The olfactory capacity for the discrimination of odors correlated inversely with the number of relapses (r = -0.5, p ≤ 0.05). The patients were aware of their olfactory deficit.

**Conclusions:**

Olfactory and gustatory dysfunction is a symptom in MS patients and may be a useful parameter to estimate disease progression in MS patients. As the discrimination of odors is processed in higher central regions of the central nervous system (CNS), the results suggest that olfactory dysfunction could be due to CNS damage.

## Introduction

Multiple Sclerosis (MS) is a chronic inflammatory disease affecting the CNS and typically follows a relapsing-remitting disease course (RRMS). Approximately 15% of all MS patients initially present with a primary progressive disease course (PPMS) characterized by constantly increasing physical disability without recovery. The neurological symptoms vary depending on the region of the brain that is affected by MS lesions [1,2]. Olfactory dysfunction mainly occurs with Alzheimer´s and Parkinson´s disease and less frequently with other neurodegenerative diseases [3,4]. Furthermore olfactory dysfunction often presents as a first symptom of Parkinson´s disease, occurring around 4–6 years before motor disorders begin [5,6]. Detecting olfactory disorders is becoming increasingly important in the research of neurological diseases [4]. A dysfunction in the olfactory sense may have a great impact on quality of life [3].

Recent studies have reported olfactory dysfunction in MS patients ranging from 11 to 41% [7–10] and gustatory dysfunction ranging from 4.5 to 19% [11–13]. In our longitudinal study we examined the olfactory and gustatory function in MS patients over a median time interval of 3 years. The aim of this study was to investigate the correlation between the olfactory and gustatory capacity and disease progression in the course of disease. To verify the olfactory test results, olfactory evoked potentials were registered for objective olfactometry.

## Materials and Methods

### Subjects

20 patients (15 women, 5 men) with diagnosed MS according to the McDonald criteria 2010 [1] were tested longitudinally over a period of 3 years. 16 patients had relapsing remitting MS, 4 patients had primary progressive MS. 14 out of 16 patients with RRMS were treated with disease-modifying drugs for MS during the testing period. 3 patients received glatiramer acetate, 3 patients interferon beta, 4 patients dimethyl fumarate, 3 patients fingolimod and 1 patient azathioprine. 2 patients with RRMS and 4 patients with primary progressive MS did not receive any immunomodulatory or immunosuppressive therapy during the testing period.

On baseline and on follow-up visit, all patients had to complete two questionnaires and underwent an otorhinolaryngological (ORL) and neurological examination. Predefined exclusion criteria were: age below 18 or over 65, pregnancy, olfactory disorders with a known different etiology (post-traumatic, post-infectious, sinonasal, infections of the upper respiratory tract, allergies, tumours treated with chemotherapy or radiation, patients suffering from depression, Parkinson´s or Alzheimer´s disease). Additionally, patients taking medications that could cause olfactory dysfunction, for example penicillamine, certain antibiotics, amitriptyline or methotrexate were excluded. Patients who had received corticosteroid treatment within the last 3 months before olfactory testing were excluded from the study since corticosteroids can affect the olfactory function [14]. The ORL examination was performed prior to the olfactory testing to exclude olfactory dysfunction caused by any other reasons than MS. A total score of at least 25 points in the Mini Mental State Examination (MMSE) had to be achieved in order to identify patients with cognitive dysfunction which was also an exclusion criterion. The Becks Depression Inventory Test (BDI) was performed to exclude patients with depression. The BDI is a self-evaluation method to detect the severity of symptoms of depression [15]. Adapted to the patients in our study we defined a score of below 15 points as an inclusion criterion. We used the Expanded Disability Status Scale (EDSS) to measure the grade of physical disability [16]. Patients with a value above 7 were excluded from the study, as they were not capable of performing all testing procedures.

Ethical approval and trial registration was obtained by the medical ethics committee of Charité, University Hospitals Berlin. Written consent was required to participate in the study.

### Olfactory testing

The Threshold Discrimination Identification Test (TDI) was used for orthonasal olfactory testing [17]. The olfactory threshold (T) was measured using 48 Sniffin’ Sticks with a 16-stage dilution series of n-butanol. The discrimination test (D) was performed with 48 Sniffin’ Sticks of different smell qualities to test the distinction of smells. Everyday odors such as “fish” or “coffee” were identified with the Identification Test (I), which consisted of 16 Sniffin’ Sticks. A TDI value of less than 16 points was rated as anosmia, up to 30.5 points as hyposmia and a value above 30.5 points as normosmia. The patients also estimated their smell function on a visual analogue scale (VAS) ranging from 0 to 10 points.

### Gustatory testing

For gustatory testing, the “Taste Strip Test” (TST) by Burghart Wedel (Germany) was used [18]. The 16-part test evaluates 4 different qualities of taste (sweet, sour, salty and bitter), each presented in 4 different concentrations (multiple forced-choice). A test value of below 9 represents a reduced sense of taste.

### Olfactory Evoked Potentials (OEP)

The OM 2/S Olfactometer (Burghart Elektro- und Feinmechanik GmbH, Germany) was used according to the guidelines of the Working Group of Olfactology and Gustology of the German ENT society [19]. A 4 mm lumen tube was placed in the nasal vestibulum and delivered an airstream which was humidified and warmed to 36.5°C with a flow of 7–8 l/min. The stimuli had a duration of 200ms and were presented in intervals between 35–40 seconds, alternating between the right and left nostril. OEPs were recorded using the international 10–20 EEG system. The olfactory stimuli consisted of phenylethyl alcohol (PEA) and hydrogen sulfide (H_2_S). For the trigeminal stimuli carbon dioxide (CO_2_) was used. The test results were interpreted according to the guidelines of the Working Group of Olfactology and Gustology of the German ENT society [19].

### Statistical analysis

Statistical analyses were performed using SPSS 23.0 (IBM SPSS Statistics, New York, USA). Graphs were created with GraphPadPrism 6.0 (GraphPad Software Inc, California, USA). The data are presented as means with standard deviations. The Wilcoxon-Man-Whitney-Test was used to compare baseline with follow-up measurements of clinical characteristics and olfactory test scores. Spearman correlations were performed to run correlational analyses on the VAS with TDI score and the number of relapses with the D-score.

## Results

### Subjects

A total of 8 patients did not perform the follow-up testing. 3 patients had to be excluded due to corticosteroid treatment they had received and 5 patients refused to perform the follow-up visit due to personal reasons. The mean EDSS-Score remained with 2 ± 2 points (p = 0.17) over the testing period. The mean number of relapses during the longitudinal study was 0,3 ± 0,5. The patients clinical characteristics are presented in Table 1.

**Table 1:** Demographics and clinical characteristics.

	Mean ± Standard Deviation	Minima / Maxima
Age	**44.9** *(41*.*8)* **± 10.1** *(10*.*1)*	**26.7***(23*.*3) /* **64 .8** *(62*.*8)*
DD	**9** *(6*.*2)* **± 7.8** *(7*.*8)*	**2** *(0) /* **33** (31)
EDSS	**2** *(2)* **± 2** *(2)*	**1** (1) / **6** *(6)*
TR	**3.6** (3.3) **± 3.7** (3.6)	**1** (1) / **16** (15)
BDI	**7** *(6)* **± 5.2** *(5)*	**0** *(0) /* **15** *(14)*
MMSE	**29** *(29)* **± 1.8** *(1)*	**25** *(27) /* **30** *(30)*

Follow up values (bold) and baseline values (brackets) [DD = Disease Duration, TR = total number of relapses, EDSS = Expanded Disability Status Scale, BDI = Becks Depression Inventory, age and DD are presented in years]. The data are presented as means with standard deviations. Sample maxima and minima are indicated.

### Olfactory testing

**Fig 1:**
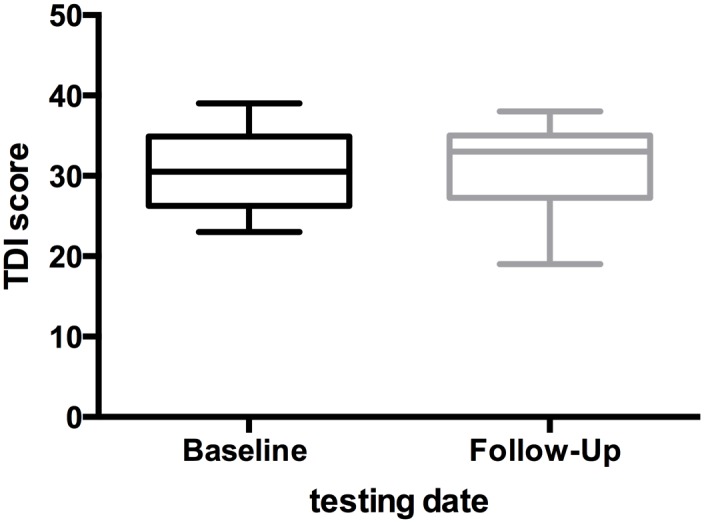
Boxplot of longitudinal TDI testing results. X-axis: time points (baseline and follow-up in years). Y-axis: TDI score (TDI = Threshold Discrimination Identification). The median is shown with first and third quartiles. The scores of the whiskers represent the minimum and maximum scores.

50% (n = 10) of the longitudinal cohort (n = 20) showed hyposmia in the baseline TDI test. In the follow-up testing 45% of the patients (n = 9) showed hyposmia. None of the patients were anosmic. Interestingly, the overall TDI score remained stable (30.6 vs 30.8 points, p = 0,78**)** over the testing period with a mean disease duration of 3.2 years. Furthermore all olfactory subscores (Threshold, Discrimination, Identification) showed no significant change during the longitudinal study. The results of the baseline and follow-up TDI measurements are presented in Table 2 and Fig 1. Olfactory raw data is presented in a supplementary table (S1 Table).

**Table 2:** Olfactory and Gustatory data.

	Mean ± Standard Deviation	Minima / Maxima
TDI	**30.8** *(30*.*6)* **± 5.7** *(5)*	**19** *(23) /* **38** (39)
T	**8** *(6*.*6)* **± 2,9** *(1*.*9)*	**3** *(3) /* **13** (10)
D	**12** *(12)* **± 2.4** *(3)*	**6** *(6) /* **16** *(15)*
I	**11** *(12)* **± 2.5** *(2)*	**4** *(8) /* **13** *(16)*
VAS	**7.7 (7) ± 1.8** (1.5)	**3** (4) / **10** (9)
TST	**10** (9.5) **± 3.1** (6)	**6** (3) / **15** (16)

Follow up values (bold) and baseline values (brackets) [TDI = Threshold Discrimination Identification, T = Threshold, D = Discrimination, I = Identification, VAS = Visual Analogue Scale, TST = Taste Strip Test]. The data are presented as means with standard deviations. Sample maxima and minima are indicated.

### Gustatory testing

20% of the patients displayed a gustatory dysfunction in the baseline measurement and in the follow-up visit. 75% of the patients with a gustatory dysfunction were also hyposmic. Gustatory test results are presented in Table 2.

### Olfactory evoked potentials

In the follow-up test, objective olfactometry was also performed. 50% of the patients showed delayed OEP´s and were classified as hyposmic. These patients had a mean TDI score of 29. The patients that were classified as normosmic in objective olfactometry had a mean TDI score of 33 points.

**Fig 2:**
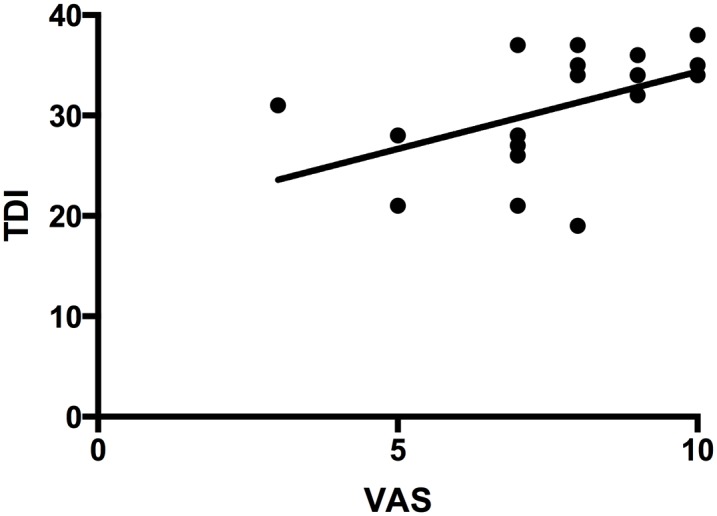
Linear correlation of TDI score with VAS. X-axis: VAS score (VAS = Visual Analogue Scale). Y-axis: TDI score (TDI = Threshold Discrimination Identification). p ≤ 0.01, r = 0.6 (Pearson correlation coefficient).

However the total number of relapses correlated with a reduced Discrimination-Score (r = -0.5, p ≤ 0.05). The VAS scale correlated with the TDI score of the longitudinally tested patients (r = 0.6 p ≤ 0.01) [Fig 2].

The patients showed mild disease activity with a mean number of 0,3 ± 0,5 relapses over the testing period and no significant change of their olfactory and gustatory capacity. Furthermore the patients showed a good self-estimation of their olfactory capacity expressed on the VAS.

## Discussion

We reported olfactory dysfunction in 45% of the longitudinally tested MS patients. Our results conform with previous studies, which reported olfactory dysfunction at rates of 11%–41% [7–10]. The olfactory capacity might have varied as different smell tests, such as the TDI test, the University of Pennsylvania Smell Identification Test (UPSIT) and Brief Smell Identification Test (B-SIT) were applied in the previous studies. Most of these studies grouped MS patients with relapsing remitting disease course and chronic progressive disease course together [7–13]. Interestingly, the olfactory and gustatory capacity of the MS patients remained stable during the longitudinal testing period in our study. The grade of physical disability expressed in the EDSS score also didn´t increase. These findings might be due to disease-modifying therapy that 70% of the patients were receiving. 75% of the patients with a gustatory dysfunction were also hyposmic. On a cortical level, the olfactory and the gustatory system intersect, in the insular cortex, the orbitofrontal cortex and the amygdala [20]. Olfactory stimuli are linked with gustatory information, delivered to the anterior insula (multimodal integration), which might explain these results.

The functional deficit in MS is caused by a demyelinating process leading to reduced conduction and inhibition in the transmission of electrophysiological impulses and secondary to an axonal degeneration [2]. A human autopsy cohort reported olfactory bulb/tract and inferior frontal cortex demyelination in 70.6% of pathologically confirmed MS cases [21]. The results from this study further support the findings of olfactory dysfunction in MS patients. Olfactory dysfunction in MS patients have been illustrated by a delayed latency and reduced amplitude of OEP´s in different studies [22, 23]. Patients showing pathological OEP´s in our study also showed olfactory dysfunction in subjective olfactory testing, which suggests a good reliability of our testing procedures. Visual, sensory, motor-evoked and acoustic-evoked potentials are clinically routine procedures used for diagnosing MS. OEP´s might serve as an additional tool in certain cases to detect a functional deficit in the olfactory pathway. Most of the MS patients showed a good self-perception of their olfactory capacity. Their self-estimation of smell was slightly higher (not significant) in the follow-up visit, which could be due to an adaptive process of the olfactory deficit. This shows that testing the olfactory capacity might be a useful additional parameter to estimate disease activity for MS patients concerning the dynamic course of their disease.

The Discrimination-Score (D-Score) was correlated with the total number of relapses during the testing period. As the discrimination of odors is processed in higher central regions of the CNS [21], this result suggests that olfactory dysfunction might occur due to CNS damage in MS patients, rather than from peripheral damage of the olfactory nerve. A different study group showed a close association longitudinally between magnetic resonance imaging (MRI) activity measured as remission and exacerbation of plaque numbers and the olfactory function in 5 patients over an 18–20 month period [24]. A correlation between the olfactory bulb volume and the olfactory capacity of patients with olfactory dysfunction with different etiologies has been demonstrated in several studies [25, 26, 27]. Our previous study showed a correlation between a decreased olfactory bulb and olfactory brain volume and increased numbers and volumes of MS lesions in the olfactory brain of MS patients [9]. These results further suggest a correlation between the olfactory function and disease activity in MS patients. Olfactory dysfunction in MS patients might be due to MS lesions or atrophy in the olfactory brain. Our study could be improved by the use of MRI to measure the lesion load and volume changes of the olfactory bulb and the olfactory brain. The application of contrast agent would have been useful to detect enhanced MS lesions and measure subclinical disease activity.

The results from this study underline the persistence of olfactory and gustatory dysfunction in MS patients. This suggests that the olfactory capacity for the discrimination of smells might be a marker for disease progression in MS patients. We reported a case of one MS patient presenting functional anosmia and gustatory dysfunction during an acute relapse; showing a significant improvement of his chemosensory function in a follow-up test one year later [28]. The incidence of olfactory dysfunction with acute relapses and improvement of olfactory function during the course of the disease suggests that acute olfactory disturbance might occur due to damage to the olfactory bulb. Given its plasticity, the olfactory bulb is able to recover from acute damage [29].

This study is possibly the first investigation into olfactory and gustatory function in more than 5 MS patients longitudinally. The patients in our study showed mild disease activity and no relevant changes in their olfactory and gustatory function. It would be interesting to perform olfactory and gustatory testing in patients with highly active MS to investigate possible increased olfactory and gustatory disturbances and the correlation to MRI activity.

## Supporting Information

S1 Table Olfactory Raw Data. S1 Table: Follow up values (bold) and baseline values (brackets) [TDI = Threshold Discrimination Identification, T = Threshold, D = Discrimination, I = Identification, PPMS = Primary Progressive Multiple Sclerosis, RRMS = Relapsing Remitting Multiple Sclerosis].
(DOCX)
