# Supplementary material for: Longitudinal Testing of Olfactory and Gustatory Function in Patients with Multiple Sclerosis
Source: PLoS One. 2017 Jan 20;12(1):e0170492. doi: 10.1371/journal.pone.0170492 (PMC5249198; doi:10.1371/journal.pone.0170492)
Supplement: S1 Table — Follow up values (bold) and baseline values (brackets) [TDI = Threshold Discrimination Identification, T = Threshold, D = Discrimination, I = Identification, PPMS = Primary Progressive Multiple Sclerosis, RRMS = Relapsing Remitting Multiple Sclerosis]. (DOCX) [file pone.0170492.s001.docx]

| **Diagnosis** | **TDI** | **T** | **D** | **I** |
| --- | --- | --- | --- | --- |
| **RRMS** | **35** (34,5) | **6** (6.5) | **16** (13) | **15** (16) |
| **RRMS** | **34** (33) | **9** (7) | **12** (15) | **13** (11) |
| **RRMS** | **37** (37) | **13** (10) | **12** (14) | **11** (13) |
| **RRMS** | **35** (36) | **12** (9) | **10** (12) | **13** (15) |
| **PPMS** | **28** (28) | **6** (5) | **11** (11) | **11** (12) |
| **RRMS** | **34** (35) | **8** (8) | **14** (15) | **12** (12) |
| **RRMS** | **31** (39) | **7** (8) | **12** (15) | **12** (16) |
| **PPMS** | **27** (29) | **5** (6) | **9** (13) | **13** (10) |
| **RRMS** | **36** (34) | **9** (8) | **14** (14) | **13** (12) |
| **RRMS** | **38** (32) | **12** (5) | **13** (12) | **13** (15) |
| **RRMS** | **37** (34) | **13** (7) | **11** (14) | **13** (13) |
| **RRMS** | **21** (26) | **4** (4) | **13** (10) | **4** (12) |
| **RRMS** | **28** (26) | **7** (5) | **10** (11) | **11** (10) |
| **RRMS** | **34** (29) | **8** (4) | **14** (11) | **12** (14) |
| **PPMS** | **32** (35) | **9** (9) | **11** (13) | **12** (14) |
| **RRMS** | **26** (23) | **4** (8) | **12** (7) | **10** (8) |
| **RRMS** | **34** (29) | **7** (6) | **16** (11) | **11** (13) |
| **RRMS** | **21** (23) | **8** (8) | **6** (6) | **7** (9) |
| **RRMS** | **28** (27) | **8** (6) | **10** (8) | **10** (13) |
| **PPMS** | **19** (23) | **3** (3) | **9** (10) | **7** (10) |
